# Prevalence and determinants of tobacco use among Iraqi adolescents: Iraq GYTS 2012

**DOI:** 10.1186/1617-9625-11-14

**Published:** 2013-06-28

**Authors:** Hamid Y Hussain, Bushra A Abdul Satar

**Affiliations:** 1 Department of Community Medicine, Faculty of Medicine, University of Baghdad, Baghdad, Iraq

**Keywords:** Adolescents, Determinants, Smoking, Prevalence, Tobacco use, GYTS

## Abstract

**Background:**

The use of any form of tobacco by 13–15 year old individuals is 10% globally as identified through the Global Youth Tobacco Survey (GYTS). This study aimed at assessing the prevalence and determinants of tobacco use among Iraqi adolescents.

**Methods:**

A cross sectional study was carried out on 1750 participants selected randomly from preparatory and secondary schools in Baghdad, Iraq in 2012. Through a multistage stratified random sample scheme. The GYTS questionnaire was applied.

**Results:**

The study results indicated that 21.8% of Iraqi adolescents are tobacco users (male 27.1%, female 12.7%). Cigarette smoking was noted as the main type of tobacco use (13.9%) followed by shisha (4.8%) and pipe (1.4%). The stepwise logistic regression indicated a number of predictors of tobacco use. Male adolescents were twice more likely to be tobacco users than female students (OR 2.31; 95%C.I: 1.57-3.42). Furthermore, students whose parents or sibling were smokers had doubled the risk of tobacco use relative to those with no parents or siblings current smokers (OR1.97; 95%C.I: 1.04-2.77 and OR1.86; 95%C.I: 1.21-2.87 respectively). Having close friends who smoked was also identified as an important risk factor towards adolescent tobacco use. Those who reported that some of their friends smoked were 2.67 times more likely to be smokers (95%C.I: 1.83-3.89), while those who reported that most/all of their friends were smokers were 8.18 times more likely to be smokers themselves (95%C.I: 4.65-14.39).

**Conclusion:**

Smoking rates among Iraqi adolescents were found to be among the higher rates of adolescent smoking prevalence in the Middle East. Multiple family and peer related characteristics were related to tobacco use. Preventive activities should take place to curb the tobacco epidemic in Iraq.

## Introduction

Adolescents are a vulnerable population in terms of health related behaviors due to their unique bio-psych-social characteristics, with tobacco experimentation a prominent behavior with potentially grave consequences, especially among adolescents in developing countries [1,2]. Tobacco use is shifting from the developed world to developing countries, especially low- and middle-income countries, such as those in the Middle East [3,4]. To date, there is limited available information on the prevalence and correlates of tobacco use among Iraqi adolescents [5]. One such study is the Kurdistan-Iraq, Global Youth Tobacco Survey which noted a prevalence of current cigarette smoking of 15.3%, while indicating the role of parental and peer smoking, male gender, increased pocket money and perceptions that boys or girls who smoked were attractive as factors related to tobacco use [5]. However, there is limited documentation of the prevalence of tobacco use in the rest of Iraq, a country that has suffered deeply from three devastating wars and long term sanctions, factors that may have affected tobacco use among Iraqi youth. With the above in mind the aim of this study was to assess the prevalence and determinants of tobacco use among the adolescent population of Bagdad, Iraq in 2012.

## Methods

A school-based analytical cross-sectional study was conducted in both preparatory and secondary schools in, Baghdad/ Iraq from 1^st^ Jan-1^st^ June, 2012. A multistage stratified random sample technique, with proportional allocation according to the number of schools was used. Stratification was based upon the regions (Two river sides regions), sex (male, female), type of school (governmental vs. private) and age categories.

The list of schools was obtained from the Ministry of Education. The selection of the private and governmental schools were carried out proportionally according to the number of schools; one public school to 4 private schools were selected to a total of 20 schools, 10 males and 10 females within each class were randomly selected from each of grade (preparatory: Years 7–9 and secondary Years:10–12). The total sample size amounted to 1951. The questionnaires of 201 students were incomplete in most items, thus were excluded, leaving a total sample size of 1750 students within the analysis.

Operational definitions for smoking and other variables were based on the World Health Organization GYTS questionnaire that has been applied within other studies [4].

Descriptive statistics were performed for quantitative variables (mean, standard deviation and range). Calculation of 95% confidence intervals around the proportions of tobacco use was performed. Chi-square test was used for testing the relationship between socio-demographic factors, knowledge, attitude and tobacco use. So as to assess the determinants of tobacco use, a stepwise logistic regression analysis was performed on the following factors: type of school, age, sex, sibling, parents, and close friends smoking, exposure to smoking in public places, attitudes and father education. Odds Ratios (OR) and 95% Confidence Intervals (95%C.I) are also presented. Data analysis was performed with the Statistical Package for Social Science (SPSS) software program version 19.0.

## Results

The study revealed that the prevalence of tobacco use among Iraqi 13–18 year olds is 21.8% out of which 13.9% smoke cigarettes, 4.8% smoke shisha, 1.3% smoke pipe, 1% smoke cigars and 0.6% use smokeless tobacco.

Table 1 presents the frequency distribution of tobacco product used by age of initiation and days of smoking within the last month. When assessing the factors related to smoking among current adolescent cigarette smokers (n = 178), we identified that the main source of obtaining tobacco was from friends (51.4%), schoolmates (19.2%) and parents (10.4%). Table 2 depicts the association between tobacco use and selected sociodemographic characteristics. Current smoking status was higher among male adolescents when compared to females, among adolescents within governmental schools and was significantly related to maternal and paternal education. Finally Table 3 presents the results of a stepwise logistic regression of the determinants of tobacco use among Iraqi adolescents. Nine significant predictors were identified and these included the type of school, age, male sex, siblings and parents that smoke, having most of the close friends smoke, being exposed exposure to smoking in public places, personal attitudes and the paternal level of education. Students aged 16 years or more had higher risk of tobacco use than those aged <16 years (OR = 2.85, 95% CI = 1.78 – 4.41). Male students were more likely to be tobacco users than females (OR 2.31; 95%C.I.: 1.57-3.42). Furthermore, students whose parents or sibling were smokers had doubled the risk of tobacco use relative to those with no parents or sibling smokers (OR1.97; 95%C.I: 1.04-2.77 and OR1.86; 95%C.I: 1.21-2.87). Having close friends who smoked was also identified as an important risk factor towards adolescent tobacco use. Specifically, those who reported that some of their friends smoked were more than twice more likely to be smokers (OR 2.67 95%C.I: 1.83-3.89), while those who reported that most/all of their friends were current smokers were 8.18 times more likely to be smokers themselves (95%C.I: 4.65-14.39).

**Table 1:** **Frequency distribution by age of initiation and days of smoking within the last month among Iraqi adolescents**, **Baghdad**, **Iraq 2012S**

	**Type of tobacco products**											
	**Cigarettes**		**Shisha**		**Pipe**		**Cigar**		**Bidi**		**Smokeless tobacco**	
	**(n = 178)**		**(n = 156**		**(n = 109)**		**(n = 44)**		**(n = 19)**		**(n = 26)**	
**Age of initiation**	N	%	N	%	N	%	N	%	N	%	N	%
**<10**	15	6.27	12	7.7	6	5.5	-	-	2	13.3	4	15.4
**10-11**	46	19.2	47	30.1	19	17.4	23	52.3	6	40.0	6	23.1
**12-13**	85	35.5	27	17.3	33	30.3	11	25.0	1	7.6	9	34.6
**14+**	93	38.9	70	44.9	51	46.8	10	22.7	6	40.0	7	26.9
**Days of tobacco use in the past 30 days**												
**1-2**	91	38.0	103	66.0	32	29.4	22	50.0	10	52.6	6	23.1
**3-5**	50	20.9	10	6.4	12	11.0	8	18.2	4	21.1	1	3.8
**5-9**	9	3.8	12	7.7	10	9.2	2	4.5	2	10.5	2	7.7
**10-19**	20	8.8	7	4.5	5	4.6	2	4.5	2	10.5	2	7.7
**20-29**	12	5.0	14	9.0	10	9.2	3	6.8	0	0	4	15.4
**Everyday**	47	19.6	10	6.4	40	36.7	7	15.9	1	5.3	11	42.3

**Table 2:** Association between tobacco use and selected sociodemographic characteristics among Iraqi adolescents, Baghdad, Iraq, 2012

**Socio demographic characteristics**	**Tobacco use**				**Total N**	**p-value**
	**yes**	**yes**	**no**	**no**		
	**N**	**%**	**N**	**%**		
School type						
governmental	124	24.21	388	75.78	512	<0.001
private	240	19.38	998	79.80	1238	
Age in Years						
<14	65	14.06	397	85.93	462	<0.001
14-16	155	26.13	438	73.68	593	
16+	144	22.32	501	77.67	645	
Gender						
male	266	27.1	508	72.9	981	<0.001
female	98	12.7	670	87.3	768	
Grade within the School						
7-9	176	22.0	622	78.0	798	<0.001
10-12	188	19.7	764	81.3	952	
Maternal education						
illiterate	104	54.7	86	45.3	190	0.003
primary	96	46.6	110	53.6	206	
preparatory	64	26.5	177	73.5	241	
secondary	52	9.8	474	90.2	526	
University/higher	48	17.7	539	82.3	587	
Paternal education						
illiterate	98	49.0	102	51.4	200	<0.001
primary	93	37.2	157	62.7	250	
secondary	80	29.6	190	70.4	270	
preparatory	73	19.1	308	79.9	381	
University/higher	60	9.2	589	90.8	649	

**Table 3:** Determinants of tobacco use among adolescents in Baghdad, Iraq 2012

**Independent variable**	**OR**	**(95% CI: Lower-Upper)**	**P-value**
**Type of school**			
Private	1.0	(0.8-1.2)	>0.05
Governmental	2.08	(1.48 - 2.93)	<0.001
**Age**			
16-18 (vs. 13-16)	2.88	(1.87 - 4.41)	<0.001
**SEX** (male)	2.31	(1.57 - 3.42)	<0.001
**PEER-Family smoking**			
Parents smoking (yes)	1.97	(1.04 - 2.77)	<0.001
Siblings smoking (yes)	1.86	(1.21 - 2.87)	0.005
**CLOSE FRIEND SMOKING**			
Some of them	2.67	(1.83 - 3.89)	<0.001
Most/ all	8.18	(4.65 - 14.39)	<0.001
**EXPOSURE TO SMOKING IN PUBLIC PLACES**			
Occasionally	1.52	(1.04 - 2.24)	0.330
Regularly	2.75	(1.62 - 4.68)	<0.001
**ATTITUDE**			
*Neutral	2.37	(1.37 - 4.12)	0.002
*Negative	3.89	(2.66-5.37)	0.000
**PATERNAL EDUCATION**			
Secondary	0.72	(0.48 - 1.80)	0.114
Primary	1.78	(1.5 - 2.75)	0.009
Illiterate	2.02	(1.15 - 4.79)	0.047

Stepwise logistic regression with type of school, age, sex, sibling, parents, close friends smoking, exposure to smoking in public places, attitudes and father education as covariates.

Neutral: The response to the attitude questions of the GYTS was neither no nor yes.

Negative: The mean response to attitude questions was negative (thus pro tobacco).

## Discussion

The current study indicated that prevalence rates of smoking among Iraq adolescents is relatively high, higher than other countries in the Middle East such as Yemen (2003) (7) and Saudi Arabia (2007) where the prevalence of any current tobacco use was 19.3% & 17.3% respectively. Moreover the smoking status of siblings, close friends and family as also the type of school was associated with tobacco use among Iraqi adolescents.

According to data presented from 75 sites in 43 countries and the Gaza Strip/West Bank region by the Global Youth Tobacco Survey (2002) the current use of any tobacco product during adolescence ranged from 3.3% to 62.8% with current cigarette smoking ranging from 1% to 39.6% [6]. Cigarettes smoking were the predominant form of tobacco use among the current tobacco users (10.7%). Our noted prevalence is similar to that reported within the last Greek GYTS study (2005) and Tehran [7,8] but it was higher than that reported by studies in Oman (2006) [9] where the prevalence of current smoking cigarettes was 4.5%.

As for the results of our multiple logistic regression analysis, we identified that type of schooling and family tobacco use was associated with smoking during adolescence, factors commonly noted as predictors of smoking in other countries [10]. Similarly to our study, the Pokhara study (2003), [9] reported that, boys were 3.15 times more likely to use tobacco compared to girls, and adolescent students from non-governmental schools were 2.58 times more likely to use tobacco than adolescent students from governmental schools. Moreover students were 1.79 times more likely to use tobacco if at least one family member (parents, siblings and other members residing permanently) used tobacco than those who had no family members using tobacco [11].

This study provides significant insight into smoking prevalence and its determinants in Iraqi adolescents, an area relatively untouched to date. However our study has a number of limitations inherent in any cross sectional school survey where data collection is limited to a single time point. Tobacco use was assessed by self-report and therefore, some students may have under reported their tobacco use, especially female students thus the estimated prevalence may be slightly lower than actual prevalence. Moreover, we did not assess other potential influential factors such as exposure to tobacco advertising and promotional activities, which have been noted to be rampant in other low and middle income countries [12].

## Conclusions

Smoking rates among Iraqi adolescents were found to be among the higher rates of adolescent smoking prevalence in the Middle East. Cigarette smoking was the most prevalent type of tobacco use, followed by shisha and pipe smoking. Multiple family and peer related characteristics were related to tobacco use. Preventive activities that could be implemented at either a community or school based level should take place to curb the tobacco epidemic in Iraq [12].

## Competing interests

The authors declared that they have no competing interests.

## Authors' contributions

Both authors read and approved the final manuscript.
